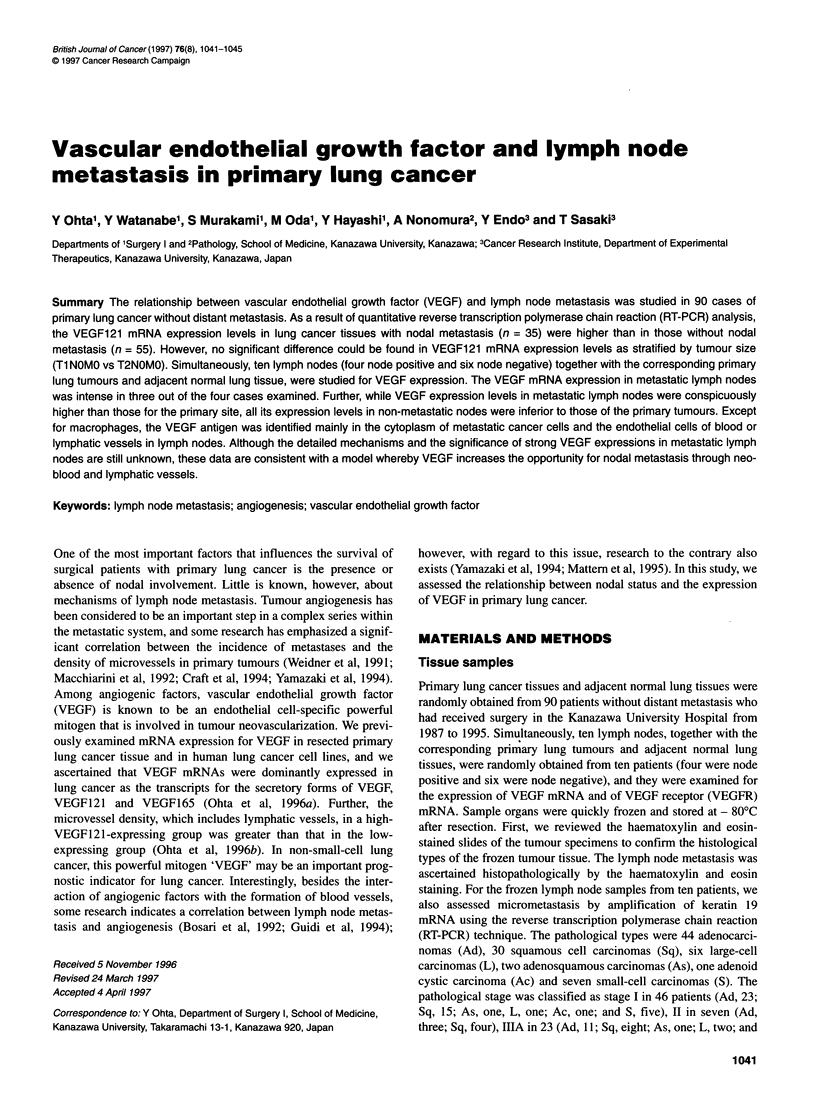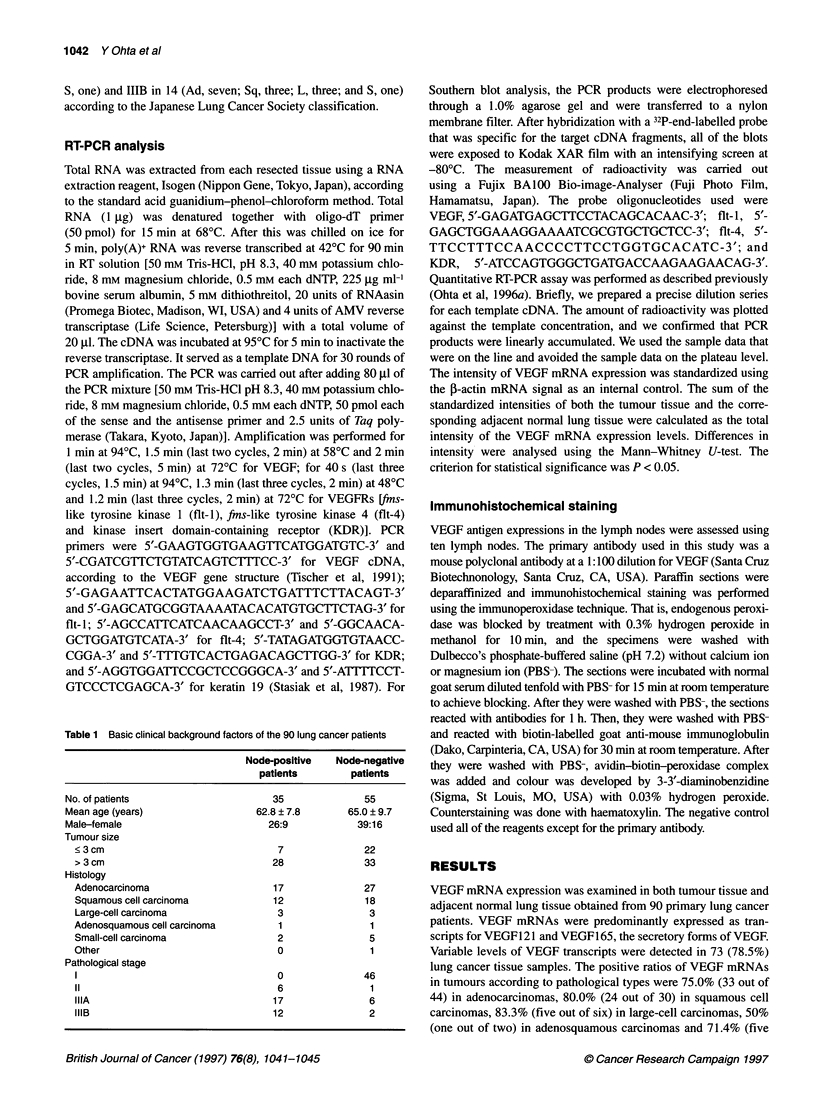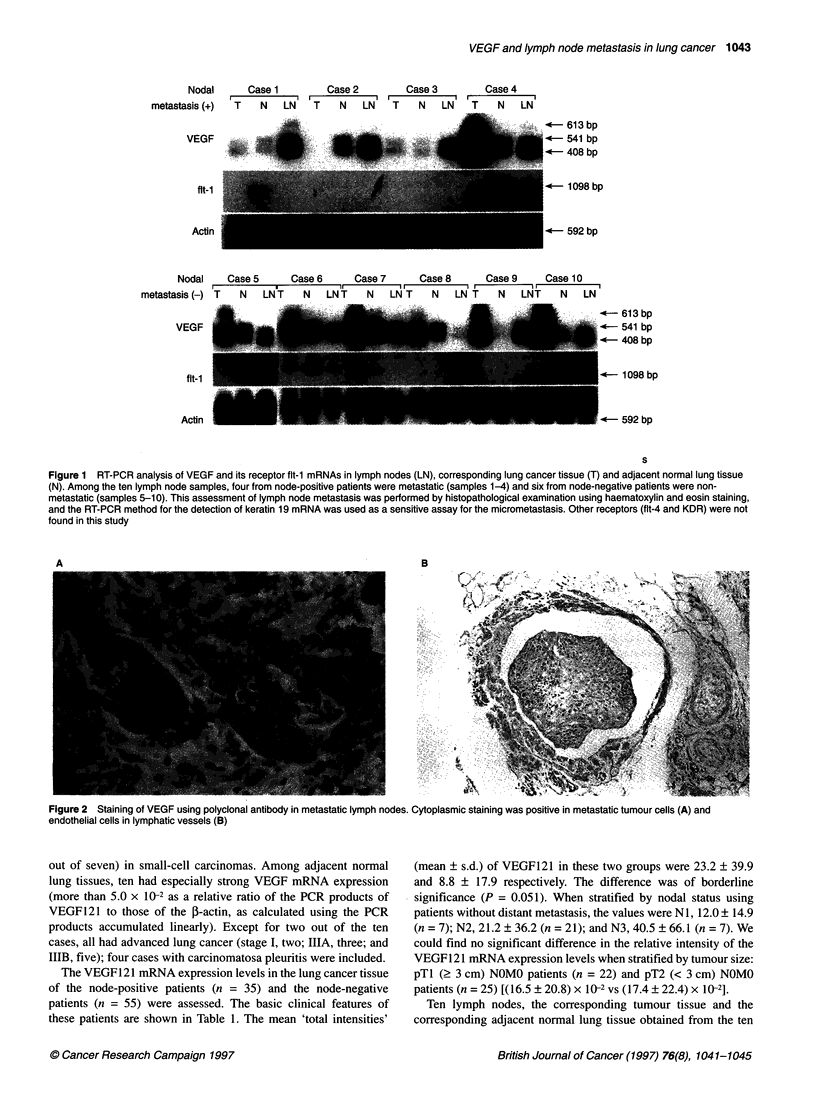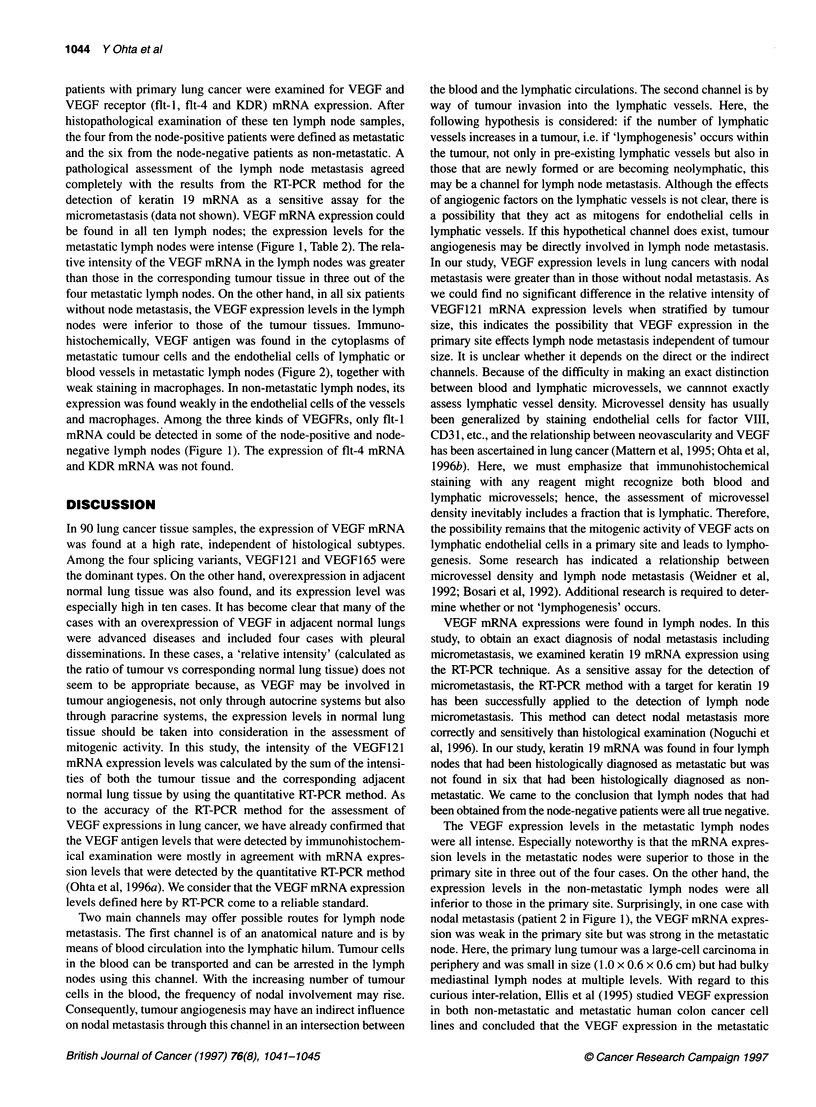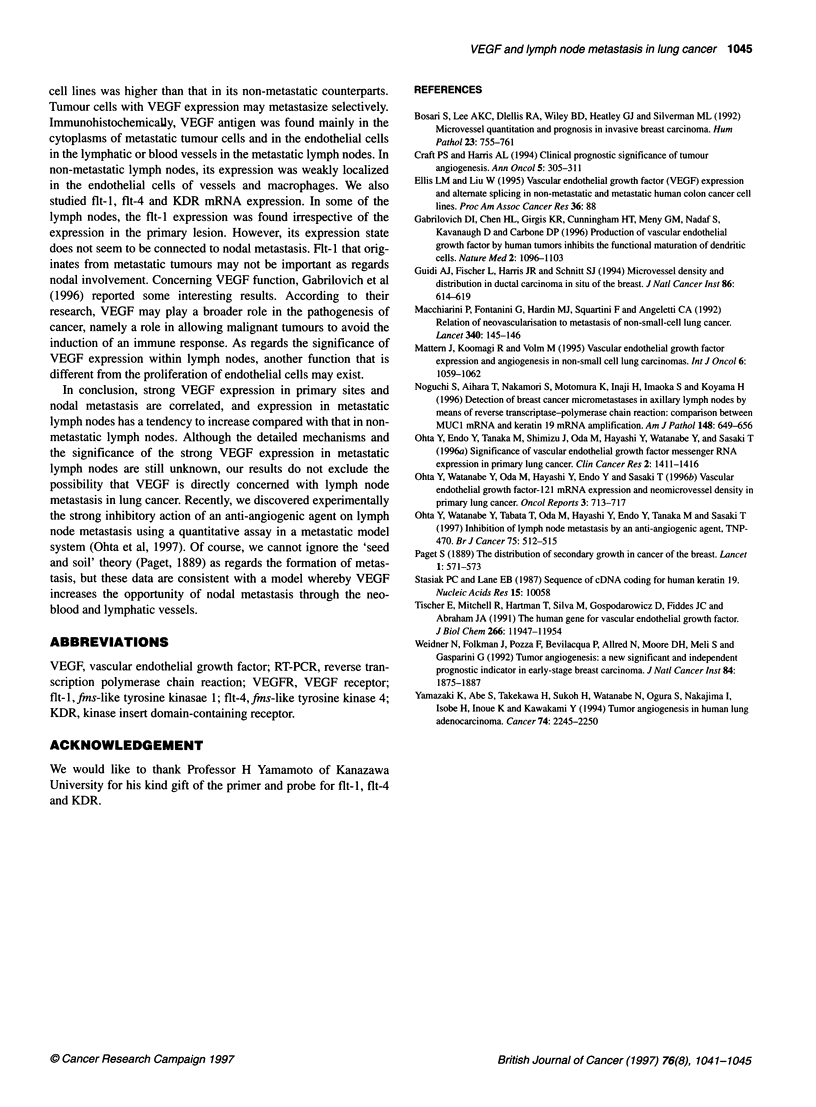# Vascular endothelial growth factor and lymph node metastasis in primary lung cancer.

**DOI:** 10.1038/bjc.1997.505

**Published:** 1997

**Authors:** Y. Ohta, Y. Watanabe, S. Murakami, M. Oda, Y. Hayashi, A. Nonomura, Y. Endo, T. Sasaki

**Affiliations:** Department of Surgery I, School of Medicine, Kanazawa University, Japan.

## Abstract

**Images:**


British Journal of Cancer (1997) 76(8), 1041–1045
© 1997 Cancer Research Campaign

### Vascular endothelial growth factor and lymph node metastasis in primary lung cancer

Y Ohta[1], Y Watanabe[1], S Murakami[1], M Oda[1], Y Hayashi[1], A Nonomura[2], Y Endo[3] and T Sasaki[3]

Departments of [1]Surgery I and [2]Pathology, School of Medicine, Kanazawa University, Kanazawa; [3]Cancer Research Institute, Department of Experimental Therapeutics, Kanazawa University, Kanazawa, Japan

**Summary** The relationship between vascular endothelial growth factor (VEGF) and lymph node metastasis was studied in 90 cases of primary lung cancer without distant metastasis. As a result of quantitative reverse transcription polymerase chain reaction (RT-PCR) analysis, the VEGF121 mRNA expression levels in lung cancer tissues with nodal metastasis ($n$ = 35) were higher than in those without nodal metastasis ($n$ = 55). However, no significant difference could be found in VEGF121 mRNA expression levels as stratified by tumour size (T1N0M0 vs T2N0M0). Simultaneously, ten lymph nodes (four node positive and six node negative) together with the corresponding primary lung tumours and adjacent normal lung tissue, were studied for VEGF expression. The VEGF mRNA expression in metastatic lymph nodes was intense in three out of the four cases examined. Further, while VEGF expression levels in metastatic lymph nodes were conspicuously higher than those for the primary site, all its expression levels in non-metastatic nodes were inferior to those of the primary tumours. Except for macrophages, the VEGF antigen was identified mainly in the cytoplasm of metastatic cancer cells and the endothelial cells of blood or lymphatic vessels in lymph nodes. Although the detailed mechanisms and the significance of strong VEGF expressions in metastatic lymph nodes are still unknown, these data are consistent with a model whereby VEGF increases the opportunity for nodal metastasis through neo-blood and lymphatic vessels.

**Keywords:** lymph node metastasis; angiogenesis; vascular endothelial growth factor

One of the most important factors that influences the survival of surgical patients with primary lung cancer is the presence or absence of nodal involvement. Little is known, however, about mechanisms of lymph node metastasis. Tumour angiogenesis has been considered to be an important step in a complex series within the metastatic system, and some research has emphasized a significant correlation between the incidence of metastases and the density of microvessels in primary tumours (Weidner et al, 1991; Macchiarini et al, 1992; Craft et al, 1994; Yamazaki et al, 1994). Among angiogenic factors, vascular endothelial growth factor (VEGF) is known to be an endothelial cell-specific powerful mitogen that is involved in tumour neovascularization. We previously examined mRNA expression for VEGF in resected primary lung cancer tissue and in human lung cancer cell lines, and we ascertained that VEGF mRNAs were dominantly expressed in lung cancer as the transcripts for the secretory forms of VEGF, VEGF121 and VEGF165 (Ohta et al, 1996*a*). Further, the microvessel density, which includes lymphatic vessels, in a high-VEGF121-expressing group was greater than that in the low-expressing group (Ohta et al, 1996*b*). In non-small-cell lung cancer, this powerful mitogen 'VEGF' may be an important prognostic indicator for lung cancer. Interestingly, besides the interaction of angiogenic factors with the formation of blood vessels, some research indicates a correlation between lymph node metastasis and angiogenesis (Bosari et al, 1992; Guidi et al, 1994); however, with regard to this issue, research to the contrary also exists (Yamazaki et al, 1994; Mattern et al, 1995). In this study, we assessed the relationship between nodal status and the expression of VEGF in primary lung cancer.

*Received 5 November 1996*
*Revised 24 March 1997*
*Accepted 4 April 1997*

*Correspondence to:* Y Ohta, Department of Surgery I, School of Medicine, Kanazawa University, Takaramachi 13-1, Kanazawa 920, Japan

### MATERIALS AND METHODS

### Tissue samples

Primary lung cancer tissues and adjacent normal lung tissues were randomly obtained from 90 patients without distant metastasis who had received surgery in the Kanazawa University Hospital from 1987 to 1995. Simultaneously, ten lymph nodes, together with the corresponding primary lung tumours and adjacent normal lung tissues, were randomly obtained from ten patients (four were node positive and six were node negative), and they were examined for the expression of VEGF mRNA and of VEGF receptor (VEGFR) mRNA. Sample organs were quickly frozen and stored at – 80°C after resection. First, we reviewed the haematoxylin and eosin-stained slides of the tumour specimens to confirm the histological types of the frozen tumour tissue. The lymph node metastasis was ascertained histopathologically by the haematoxylin and eosin staining. For the frozen lymph node samples from ten patients, we also assessed micrometastasis by amplification of keratin 19 mRNA using the reverse transcription polymerase chain reaction (RT-PCR) technique. The pathological types were 44 adenocarcinomas (Ad), 30 squamous cell carcinomas (Sq), six large-cell carcinomas (L), two adenosquamous carcinomas (As), one adenoid cystic carcinoma (Ac) and seven small-cell carcinomas (S). The pathological stage was classified as stage I in 46 patients (Ad, 23; Sq, 15; As, one, L, one; Ac, one; and S, five), II in seven (Ad, three; Sq, four), IIIA in 23 (Ad, 11; Sq, eight; As, one; L, two; and

S, one) and IIIB in 14 (Ad, seven; Sq, three; L, three; and S, one) according to the Japanese Lung Cancer Society classification.

### RT-PCR analysis

Total RNA was extracted from each resected tissue using a RNA extraction reagent, Isogen (Nippon Gene, Tokyo, Japan), according to the standard acid guanidium–phenol–chloroform method. Total RNA (1 μg) was denatured together with oligo-dT primer (50 pmol) for 15 min at 68°C. After this was chilled on ice for 5 min, poly(A)$^+$ RNA was reverse transcribed at 42°C for 90 min in RT solution [50 mM Tris-HCl, pH 8.3, 40 mM potassium chloride, 8 mM magnesium chloride, 0.5 mM each dNTP, 225 μg ml$^{-1}$ bovine serum albumin, 5 mM dithiothreitol, 20 units of RNAasin (Promega Biotec, Madison, WI, USA) and 4 units of AMV reverse transcriptase (Life Science, Petersburg)] with a total volume of 20 μl. The cDNA was incubated at 95°C for 5 min to inactivate the reverse transcriptase. It served as a template DNA for 30 rounds of PCR amplification. The PCR was carried out after adding 80 μl of the PCR mixture [50 mM Tris-HCl pH 8.3, 40 mM potassium chloride, 8 mM magnesium chloride, 0.5 mM each dNTP, 50 pmol each of the sense and the antisense primer and 2.5 units of *Taq* polymerase (Takara, Kyoto, Japan)]. Amplification was performed for 1 min at 94°C, 1.5 min (last two cycles, 2 min) at 58°C and 2 min (last two cycles, 5 min) at 72°C for VEGF; for 40 s (last three cycles, 1.5 min) at 94°C, 1.3 min (last three cycles, 2 min) at 48°C and 1.2 min (last three cycles, 2 min) at 72°C for VEGFRs [*fms*-like tyrosine kinase 1 (flt-1), *fms*-like tyrosine kinase 4 (flt-4) and kinase insert domain-containing receptor (KDR)]. PCR primers were 5′-GAAGTGGTGAAGTTCATGGATGTC-3′ and 5′-CGATCGTTCTGTATCAGTCTTTCC-3′ for VEGF cDNA, according to the VEGF gene structure (Tischer et al, 1991); 5′-GAGAATTCACTATGGAAGATCTGATTTCTTACAGT-3′ and 5′-GAGCATGCGGTAAAATACACATGTGCTTCTAG-3′ for flt-1; 5′-AGCCATTCATCAACAAGCCT-3′ and 5′-GGCAACAGCTGGATGTCATA-3′ for flt-4; 5′-TATAGATGGTGTAACCCGGA-3′ and 5′-TTTGTCACTGAGACAGCTTGG-3′ for KDR; and 5′-AGGTGGATTCCGCTCCGGGCA-3′ and 5′-ATTTTCCTGTCCCTCGAGCA-3′ for keratin 19 (Stasiak et al, 1987). For Southern blot analysis, the PCR products were electrophoresed through a 1.0% agarose gel and were transferred to a nylon membrane filter. After hybridization with a $^{32}$P-end-labelled probe that was specific for the target cDNA fragments, all of the blots were exposed to Kodak XAR film with an intensifying screen at –80°C. The measurement of radioactivity was carried out using a Fujix BA100 Bio-image-Analyser (Fuji Photo Film, Hamamatsu, Japan). The probe oligonucleotides used were VEGF, 5′-GAGATGAGCTTCCTACAGCACAAC-3′; flt-1, 5′-GAGCTGGAAAGGAAAATCGCGTGCTGCTCC-3′; flt-4, 5′-TTCCTTTCCAACCCCTTCCTGGTGCACATC-3′; and KDR, 5′-ATCCAGTGGGCTGATGACCAAGAAGAACAG-3′. Quantitative RT-PCR assay was performed as described previously (Ohta et al, 1996*a*). Briefly, we prepared a precise dilution series for each template cDNA. The amount of radioactivity was plotted against the template concentration, and we confirmed that PCR products were linearly accumulated. We used the sample data that were on the line and avoided the sample data on the plateau level. The intensity of VEGF mRNA expression was standardized using the β-actin mRNA signal as an internal control. The sum of the standardized intensities of both the tumour tissue and the corresponding adjacent normal lung tissue were calculated as the total intensity of the VEGF mRNA expression levels. Differences in intensity were analysed using the Mann–Whitney *U*-test. The criterion for statistical significance was $P < 0.05$.

**Table 1** Basic clinical background factors of the 90 lung cancer patients

| | Node-positive patients | Node-negative patients |
|---|---|---|
| No. of patients | 35 | 55 |
| Mean age (years) | 62.8 ± 7.8 | 65.0 ± 9.7 |
| Male–female | 26:9 | 39:16 |
| Tumour size | | |
| ≤ 3 cm | 7 | 22 |
| > 3 cm | 28 | 33 |
| Histology | | |
| Adenocarcinoma | 17 | 27 |
| Squamous cell carcinoma | 12 | 18 |
| Large-cell carcinoma | 3 | 3 |
| Adenosquamous cell carcinoma | 1 | 1 |
| Small-cell carcinoma | 2 | 5 |
| Other | 0 | 1 |
| Pathological stage | | |
| I | 0 | 46 |
| II | 6 | 1 |
| IIIA | 17 | 6 |
| IIIB | 12 | 2 |

### Immunohistochemical staining

VEGF antigen expressions in the lymph nodes were assessed using ten lymph nodes. The primary antibody used in this study was a mouse polyclonal antibody at a 1:100 dilution for VEGF (Santa Cruz Biotechnonology, Santa Cruz, CA, USA). Paraffin sections were deparaffinized and immunohistochemical staining was performed using the immunoperoxidase technique. That is, endogenous peroxidase was blocked by treatment with 0.3% hydrogen peroxide in methanol for 10 min, and the specimens were washed with Dulbecco's phosphate-buffered saline (pH 7.2) without calcium ion or magnesium ion (PBS$^-$). The sections were incubated with normal goat serum diluted tenfold with PBS$^-$ for 15 min at room temperature to achieve blocking. After they were washed with PBS$^-$, the sections reacted with antibodies for 1 h. Then, they were washed with PBS$^-$ and reacted with biotin-labelled goat anti-mouse immunoglobulin (Dako, Carpinteria, CA, USA) for 30 min at room temperature. After they were washed with PBS$^-$, avidin–biotin–peroxidase complex was added and colour was developed by 3-3′-diaminobenzidine (Sigma, St Louis, MO, USA) with 0.03% hydrogen peroxide. Counterstaining was done with haematoxylin. The negative control used all of the reagents except for the primary antibody.

### RESULTS

VEGF mRNA expression was examined in both tumour tissue and adjacent normal lung tissue obtained from 90 primary lung cancer patients. VEGF mRNAs were predominantly expressed as transcripts for VEGF121 and VEGF165, the secretory forms of VEGF. Variable levels of VEGF transcripts were detected in 73 (78.5%) lung cancer tissue samples. The positive ratios of VEGF mRNAs in tumours according to pathological types were 75.0% (33 out of 44) in adenocarcinomas, 80.0% (24 out of 30) in squamous cell carcinomas, 83.3% (five out of six) in large-cell carcinomas, 50% (one out of two) in adenosquamous carcinomas and 71.4% (five

© Cancer Research Campaign 1997

**Figure 1** RT-PCR analysis of VEGF and its receptor flt-1 mRNAs in lymph nodes (LN), corresponding lung cancer tissue (T) and adjacent normal lung tissue (N). Among the ten lymph node samples, four from node-positive patients were metastatic (samples 1–4) and six from node-negative patients were non-metastatic (samples 5–10). This assessment of lymph node metastasis was performed by histopathological examination using haematoxylin and eosin staining, and the RT-PCR method for the detection of keratin 19 mRNA was used as a sensitive assay for the micrometastasis. Other receptors (flt-4 and KDR) were not found in this study

**Figure 2** Staining of VEGF using polyclonal antibody in metastatic lymph nodes. Cytoplasmic staining was positive in metastatic tumour cells (**A**) and endothelial cells in lymphatic vessels (**B**)

out of seven) in small-cell carcinomas. Among adjacent normal lung tissues, ten had especially strong VEGF mRNA expression (more than $5.0 \times 10^{-2}$ as a relative ratio of the PCR products of VEGF121 to those of the β-actin, as calculated using the PCR products accumulated linearly). Except for two out of the ten cases, all had advanced lung cancer (stage I, two; IIIA, three; and IIIB, five); four cases with carcinomatosa pleuritis were included.

The VEGF121 mRNA expression levels in the lung cancer tissue of the node-positive patients ($n$ = 35) and the node-negative patients ($n$ = 55) were assessed. The basic clinical features of these patients are shown in Table 1. The mean 'total intensities' (mean ± s.d.) of VEGF121 in these two groups were 23.2 ± 39.9 and 8.8 ± 17.9 respectively. The difference was of borderline significance ($P$ = 0.051). When stratified by nodal status using patients without distant metastasis, the values were N1, 12.0 ± 14.9 ($n$ = 7); N2, 21.2 ± 36.2 ($n$ = 21); and N3, 40.5 ± 66.1 ($n$ = 7). We could find no significant difference in the relative intensity of the VEGF121 mRNA expression levels when stratified by tumour size: pT1 (≥ 3 cm) N0M0 patients ($n$ = 22) and pT2 (< 3 cm) N0M0 patients ($n$ = 25) [$(16.5 \pm 20.8) \times 10^{-2}$ vs $(17.4 \pm 22.4) \times 10^{-2}$].

Ten lymph nodes, the corresponding tumour tissue and the corresponding adjacent normal lung tissue obtained from the ten

© Cancer Research Campaign 1997

patients with primary lung cancer were examined for VEGF and VEGF receptor (flt-1, flt-4 and KDR) mRNA expression. After histopathological examination of these ten lymph node samples, the four from the node-positive patients were defined as metastatic and the six from the node-negative patients as non-metastatic. A pathological assessment of the lymph node metastasis agreed completely with the results from the RT-PCR method for the detection of keratin 19 mRNA as a sensitive assay for the micrometastasis (data not shown). VEGF mRNA expression could be found in all ten lymph nodes; the expression levels for the metastatic lymph nodes were intense (Figure 1, Table 2). The relative intensity of the VEGF mRNA in the lymph nodes was greater than those in the corresponding tumour tissue in three out of the four metastatic lymph nodes. On the other hand, in all six patients without node metastasis, the VEGF expression levels in the lymph nodes were inferior to those of the tumour tissues. Immunohistochemically, VEGF antigen was found in the cytoplasms of metastatic tumour cells and the endothelial cells of lymphatic or blood vessels in metastatic lymph nodes (Figure 2), together with weak staining in macrophages. In non-metastatic lymph nodes, its expression was found weakly in the endothelial cells of the vessels and macrophages. Among the three kinds of VEGFRs, only flt-1 mRNA could be detected in some of the node-positive and node-negative lymph nodes (Figure 1). The expression of flt-4 mRNA and KDR mRNA was not found.

### DISCUSSION

In 90 lung cancer tissue samples, the expression of VEGF mRNA was found at a high rate, independent of histological subtypes. Among the four splicing variants, VEGF121 and VEGF165 were the dominant types. On the other hand, overexpression in adjacent normal lung tissue was also found, and its expression level was especially high in ten cases. It has become clear that many of the cases with an overexpression of VEGF in adjacent normal lungs were advanced diseases and included four cases with pleural disseminations. In these cases, a 'relative intensity' (calculated as the ratio of tumour vs corresponding normal lung tissue) does not seem to be appropriate because, as VEGF may be involved in tumour angiogenesis, not only through autocrine systems but also through paracrine systems, the expression levels in normal lung tissue should be taken into consideration in the assessment of mitogenic activity. In this study, the intensity of the VEGF121 mRNA expression levels was calculated by the sum of the intensities of both the tumour tissue and the corresponding adjacent normal lung tissue by using the quantitative RT-PCR method. As to the accuracy of the RT-PCR method for the assessment of VEGF expressions in lung cancer, we have already confirmed that the VEGF antigen levels that were detected by immunohistochemical examination were mostly in agreement with mRNA expression levels that were detected by the quantitative RT-PCR method (Ohta et al, 1996*a*). We consider that the VEGF mRNA expression levels defined here by RT-PCR come to a reliable standard.

Two main channels may offer possible routes for lymph node metastasis. The first channel is of an anatomical nature and is by means of blood circulation into the lymphatic hilum. Tumour cells in the blood can be transported and can be arrested in the lymph nodes using this channel. With the increasing number of tumour cells in the blood, the frequency of nodal involvement may rise. Consequently, tumour angiogenesis may have an indirect influence on nodal metastasis through this channel in an intersection between the blood and the lymphatic circulations. The second channel is by way of tumour invasion into the lymphatic vessels. Here, the following hypothesis is considered: if the number of lymphatic vessels increases in a tumour, i.e. if 'lymphogenesis' occurs within the tumour, not only in pre-existing lymphatic vessels but also in those that are newly formed or are becoming neolymphatic, this may be a channel for lymph node metastasis. Although the effects of angiogenic factors on the lymphatic vessels is not clear, there is a possibility that they act as mitogens for endothelial cells in lymphatic vessels. If this hypothetical channel does exist, tumour angiogenesis may be directly involved in lymph node metastasis. In our study, VEGF expression levels in lung cancers with nodal metastasis were greater than in those without nodal metastasis. As we could find no significant difference in the relative intensity of VEGF121 mRNA expression levels when stratified by tumour size, this indicates the possibility that VEGF expression in the primary site effects lymph node metastasis independent of tumour size. It is unclear whether it depends on the direct or the indirect channels. Because of the difficulty in making an exact distinction between blood and lymphatic microvessels, we cannnot exactly assess lymphatic vessel density. Microvessel density has usually been generalized by staining endothelial cells for factor VIII, CD31, etc., and the relationship between neovascularity and VEGF has been ascertained in lung cancer (Mattern et al, 1995; Ohta et al, 1996*b*). Here, we must emphasize that immunohistochemical staining with any reagent might recognize both blood and lymphatic microvessels; hence, the assessment of microvessel density inevitably includes a fraction that is lymphatic. Therefore, the possibility remains that the mitogenic activity of VEGF acts on lymphatic endothelial cells in a primary site and leads to lymphogenesis. Some research has indicated a relationship between microvessel density and lymph node metastasis (Weidner et al, 1992; Bosari et al, 1992). Additional research is required to determine whether or not 'lymphogenesis' occurs.

VEGF mRNA expressions were found in lymph nodes. In this study, to obtain an exact diagnosis of nodal metastasis including micrometastasis, we examined keratin 19 mRNA expression using the RT-PCR technique. As a sensitive assay for the detection of micrometastasis, the RT-PCR method with a target for keratin 19 has been successfully applied to the detection of lymph node micrometastasis. This method can detect nodal metastasis more correctly and sensitively than histological examination (Noguchi et al, 1996). In our study, keratin 19 mRNA was found in four lymph nodes that had been histologically diagnosed as metastatic but was not found in six that had been histologically diagnosed as non-metastatic. We came to the conclusion that lymph nodes that had been obtained from the node-negative patients were all true negative.

The VEGF expression levels in the metastatic lymph nodes were all intense. Especially noteworthy is that the mRNA expression levels in the metastatic nodes were superior to those in the primary site in three out of the four cases. On the other hand, the expression levels in the non-metastatic lymph nodes were all inferior to those in the primary site. Surprisingly, in one case with nodal metastasis (patient 2 in Figure 1), the VEGF mRNA expression was weak in the primary site but was strong in the metastatic node. Here, the primary lung tumour was a large-cell carcinoma in periphery and was small in size ($1.0 \times 0.6 \times 0.6$ cm) but had bulky mediastinal lymph nodes at multiple levels. With regard to this curious inter-relation, Ellis et al (1995) studied VEGF expression in both non-metastatic and metastatic human colon cancer cell lines and concluded that the VEGF expression in the metastatic

© Cancer Research Campaign 1997

cell lines was higher than that in its non-metastatic counterparts. Tumour cells with VEGF expression may metastasize selectively. Immunohistochemically, VEGF antigen was found mainly in the cytoplasms of metastatic tumour cells and in the endothelial cells in the lymphatic or blood vessels in the metastatic lymph nodes. In non-metastatic lymph nodes, its expression was weakly localized in the endothelial cells of vessels and macrophages. We also studied flt-1, flt-4 and KDR mRNA expression. In some of the lymph nodes, the flt-1 expression was found irrespective of the expression in the primary lesion. However, its expression state does not seem to be connected to nodal metastasis. Flt-1 that originates from metastatic tumours may not be important as regards nodal involvement. Concerning VEGF function, Gabrilovich et al (1996) reported some interesting results. According to their research, VEGF may play a broader role in the pathogenesis of cancer, namely a role in allowing malignant tumours to avoid the induction of an immune response. As regards the significance of VEGF expression within lymph nodes, another function that is different from the proliferation of endothelial cells may exist.

In conclusion, strong VEGF expression in primary sites and nodal metastasis are correlated, and expression in metastatic lymph nodes has a tendency to increase compared with that in non-metastatic lymph nodes. Although the detailed mechanisms and the significance of the strong VEGF expression in metastatic lymph nodes are still unknown, our results do not exclude the possibility that VEGF is directly concerned with lymph node metastasis in lung cancer. Recently, we discovered experimentally the strong inhibitory action of an anti-angiogenic agent on lymph node metastasis using a quantitative assay in a metastatic model system (Ohta et al, 1997). Of course, we cannot ignore the 'seed and soil' theory (Paget, 1889) as regards the formation of metastasis, but these data are consistent with a model whereby VEGF increases the opportunity of nodal metastasis through the neo-blood and lymphatic vessels.

### ABBREVIATIONS

VEGF, vascular endothelial growth factor; RT-PCR, reverse transcription polymerase chain reaction; VEGFR, VEGF receptor; flt-1, *fms*-like tyrosine kinasae 1; flt-4, *fms*-like tyrosine kinase 4; KDR, kinase insert domain-containing receptor.

### ACKNOWLEDGEMENT

We would like to thank Professor H Yamamoto of Kanazawa University for his kind gift of the primer and probe for flt-1, flt-4 and KDR.

### REFERENCES

Bosari S, Lee AKC, Dlellis RA, Wiley BD, Heatley GJ and Silverman ML (1992) Microvessel quantitation and prognosis in invasive breast carcinoma. *Hum Pathol* **23**: 755–761

Craft PS and Harris AL (1994) Clinical prognostic significance of tumour angiogenesis. *Ann Oncol* **5**: 305–311

Ellis LM and Liu W (1995) Vascular endothelial growth factor (VEGF) expression and alternate splicing in non-metastatic and metastatic human colon cancer cell lines. *Proc Am Assoc Cancer Res* **36**: 88

Gabrilovich DI, Chen HL, Girgis KR, Cunningham HT, Meny GM, Nadaf S, Kavanaugh D and Carbone DP (1996) Production of vascular endothelial growth factor by human tumors inhibits the functional maturation of dendritic cells. *Nature Med* **2**: 1096–1103

Guidi AJ, Fischer L, Harris JR and Schnitt SJ (1994) Microvessel density and distribution in ductal carcinoma in situ of the breast. *J Natl Cancer Inst* **86**: 614–619

Macchiarini P, Fontanini G, Hardin MJ, Squartini F and Angeletti CA (1992) Relation of neovascularisation to metastasis of non-small-cell lung cancer. *Lancet* **340**: 145–146

Mattern J, Koomagi R and Volm M (1995) Vascular endothelial growth factor expression and angiogenesis in non-small cell lung carcinomas. *Int J Oncol* **6**: 1059–1062

Noguchi S, Aihara T, Nakamori S, Motomura K, Inaji H, Imaoka S and Koyama H (1996) Detection of breast cancer micrometastases in axillary lymph nodes by means of reverse transcriptase–polymerase chain reaction: comparison between MUC1 mRNA and keratin 19 mRNA amplification. *Am J Pathol* **148**: 649–656

Ohta Y, Endo Y, Tanaka M, Shimizu J, Oda M, Hayashi Y, Watanabe Y, and Sasaki T (1996*a*) Significance of vascular endothelial growth factor messenger RNA expression in primary lung cancer. *Clin Cancer Res* **2**: 1411–1416

Ohta Y, Watanabe Y, Oda M, Hayashi Y, Endo Y and Sasaki T (1996*b*) Vascular endothelial growth factor-121 mRNA expression and neomicrovessel density in primary lung cancer. *Oncol Reports* **3**: 713–717

Ohta Y, Watanabe Y, Tabata T, Oda M, Hayashi Y, Endo Y, Tanaka M and Sasaki T (1997) Inhibition of lymph node metastasis by an anti-angiogenic agent, TNP-470. *Br J Cancer* 75: 512–515

Paget S (1889) The distribution of secondary growth in cancer of the breast. *Lancet* **1**: 571–573

Stasiak PC and Lane EB (1987) Sequence of cDNA coding for human keratin 19. *Nucleic Acids Res* **15**: 10058

Tischer E, Mitchell R, Hartman T, Silva M, Gospodarowicz D, Fiddes JC and Abraham JA (1991) The human gene for vascular endothelial growth factor. *J Biol Chem* **266**: 11947–11954

Weidner N, Folkman J, Pozza F, Bevilacqua P, Allred N, Moore DH, Meli S and Gasparini G (1992) Tumor angiogenesis: a new significant and independent prognostic indicator in early-stage breast carcinoma. *J Natl Cancer Inst* **84**: 1875–1887

Yamazaki K, Abe S, Takekawa H, Sukoh H, Watanabe N, Ogura S, Nakajima I, Isobe H, Inoue K and Kawakami Y (1994) Tumor angiogenesis in human lung adenocarcinoma. *Cancer* **74**: 2245–2250

© Cancer Research Campaign 1997